# Prevalence of Binge Eating Behavior Among High School Students and Associations with Social Media Use, Mental Health, and Suicide Risk — Youth Risk Behavior Survey, United States, 2025

**DOI:** 10.15585/mmwr.su7501a5

**Published:** 2026-10-15

**Authors:** Ryan Saelee, Ariel Beccia, Brittany Celebrano, S. Bryn Austin, Allison Ivie, Simone C. Gray, Emily Young, Eric G. Carbone, Carmen Ashley, Emiko Petrosky, Jonetta J. Mpofu

**Affiliations:** ^1^Division of Adolescent and School Health, National Center for Chronic Disease Prevention and Health Promotion, CDC, Atlanta, Georgia; ^2^Division of Adolescent and Young Adult Medicine, Boston Children's Hospital, Boston, Massachusetts; ^3^Department of Pediatrics, Harvard Medical School, Boston, Massachusetts; ^4^Department of Epidemiology, Harvard T. H. Chan School of Public Health, Boston, Massachusetts; ^5^Bureau of Nutrition and Physical Activity, Arizona Department of Health Services, Phoenix, Arizona; ^6^Department of Social and Behavioral Sciences, Harvard T. H. Chan School of Public Health, Boston, Massachusetts; ^7^Eating Disorders Coalition for Research, Policy, and Action, Washington, DC; ^8^Division of Injury Prevention, National Center for Injury Prevention and Control, CDC, Atlanta, Georgia; ^9^U.S. Public Health Service, Rockville, Maryland

## Abstract

Disordered eating among adolescents is a public health concern, and binge eating, which is eating an unusually large amount of food while feeling a loss of control, is among the most prevalent forms. In 2025, for the first time, the nationally representative Youth Risk Behavior Survey (YRBS) included an item assessing binge eating among U.S. high school students. Among students with binge eating information available (N = 6,898), this report describes the first national prevalence estimates of binge eating overall and by demographic characteristics and examines associations between binge eating and social media use, mental health, and suicide risk. Pairwise *t* tests were used to identify demographic differences, and logistic regression models with predicted marginals were used to estimate adjusted prevalence ratios to compare binge eating prevalence by social media use, mental health, and suicide risk, overall and by sex. During 2025, 40.5% of U.S. high school students reported binge eating at least 1 day in the past 30 days, with 17.2% reporting ≥3 days. Prevalence of binge eating on ≥3 days was higher among female (18.6%) than male students (15.9%); higher among Hispanic (20.5%) and Black or African American (18.7%) than White (14.6%) and Middle Eastern or North African (9.9%) students; and higher among multiracial (19.6%) than Middle Eastern or North African students. Overall, students who reported using social media hourly or more, having poor mental health, having persistent sadness or hopelessness, or having seriously considered attempting suicide had a higher prevalence of binge eating on ≥3 days than those who did not report these factors. These associations between binge eating and social media use, mental health, and suicide risk were observed among both female and male students. The findings might guide efforts to expand binge eating surveillance among adolescents and identify correlates that could support surveillance and future intervention research.

## Introduction

Eating disorders, including anorexia nervosa, bulimia nervosa, and binge eating disorder, are serious diagnosed mental illnesses characterized by meaningful disturbances in eating patterns and, often, body image (*1*). Initial onset of eating disorders occurs primarily during adolescence and young adulthood (*1*). Disordered eating, which is defined as eating- and weight-related behaviors such as fasting, purging, and binge eating that might negatively affect health but do not necessarily meet diagnostic criteria for an eating disorder, might be common, with estimates varying depending on the measure and population studied. In a systematic review and meta-analysis using the Sick, Control, One, Fat, Food (SCOFF) screening tool, estimates of disordered eating ranged from 5.6% to 20.5% across studies with different samples of U.S. children and adolescents (*2*). Separately, 29.1% of Arizona high school students reported unhealthy weight-control behaviors in the 2021 Arizona Youth Risk Behavior Survey (*3*). These behaviors might be symptoms, correlates, or markers of eating-disorder risk and are associated with adverse health outcomes in adolescent and young adult populations. Among adolescents, disordered eating behaviors have been associated with depressive symptoms and lower self-esteem (*4*), whereas among young adults, these behaviors have been associated with poor self-rated health and psychological distress (*5*).

Binge eating is a common form of disordered eating among youths and involves eating an unusually large amount of food while feeling a loss of control (*2*,*4*). As with all forms of disordered eating, binge eating might occur without meeting the frequency, duration, severity, or other criteria required for a diagnosis of binge eating disorder. Previous research found that among adolescents, binge eating behaviors varied by sociodemographic characteristics, including age, sex, race and ethnicity, and household income (*6*). Binge eating also has been associated with psychological distress, including depression, anxiety, suicidal ideation, and suicide attempts (*7*,*8*). Taken together, these associations suggest that binge eating might be an important consideration within the broader youth mental health crisis (*9*).

Social media might be another important factor to consider in relation to binge eating given its widespread use among adolescents; in 2023, 77.0% of U.S. high school students reported using social media at least several times per day (*10*,*11*). More recent national data indicate that teens continue to use multiple social media platforms frequently, with overall usage patterns remaining relatively stable since 2022 (*12*). A growing body of evidence suggests that social media use might be related to binge eating through greater exposure to appearance- and diet-related content, social comparison, and the internalization of idealized body standards, all of which have been proposed as possible pathways to body dissatisfaction and can result in cycles of dietary restriction and binge eating (*10*).

Using 2025 Youth Risk Behavior Survey (YRBS) data, this report estimates the prevalence of binge eating among U.S. high school students, identifies disproportionately affected groups, and examines associations between binge eating and social media use, mental health, and suicide risk. This is the first YRBS since the 2013 survey that CDC has collected nationally representative data on any disordered eating behaviors among youths. The 2025 survey was also the first YRBS to capture data specifically on binge eating, providing the first national estimates of binge eating behavior among U.S. high school students. This survey addition was made in response to growing interest from partners in the field (e.g., researchers, public health practitioners, and school and education professionals) and used the flexibility of the YRBS system in capturing data on emerging adolescent health behaviors and experiences. The findings in this report are intended to guide these partners’ efforts to expand surveillance of binge eating among adolescents and identify groups and factors to consider for future intervention research and surveillance planning.

## Methods

### Data Source

This report includes data from the 2025 YRBS (N = 10,710), a cross-sectional, school-based survey conducted biennially since 1991. Each survey year, CDC collects data from a nationally representative sample of students from public and private schools, including parochial schools, in grades 9–12 in the 50 U.S. states and the District of Columbia. Additional information about YRBS sampling, data collection, response rates, and processing is available in the overview report of this supplement (*13*). The prevalence estimates for binge eating and other YRBS variables among U.S. high school students overall and stratified by demographic characteristics are available at https://youthonline.cdc.gov. The full YRBS questionnaire, datasets, and documentation are available at https://www.cdc.gov/yrbs/index.html. This study was approved by institutional review boards at CDC and ICF, the survey contractor, and was conducted consistent with applicable federal law and CDC policy.*

### Measures

Questions, response options, and analytic coding for binge eating and the other health behaviors and experiences examined are provided (Table 1). Information for binge eating was obtained from responses to the question, “During the past 30 days, on how many days did you eat an unusually large amount of food in a short period of time and experience a loss of control over how much you were eating or a feeling that you could not stop eating even when full?” Responses were dichotomized as binge eating on ≥3 days in the past 30 days (3–5 days, 6–9 days​, 10–19 days​, 20–29 days​, or all 30 days) versus <3 days (0 days or 1 or 2 days). The threshold of ≥3 days was selected as a conservative analytic approach intended to distinguish recurrent binge eating from isolated overeating when using a single self-report item. Of the 10,710 students in the analytic sample, 3,501 were not administered the binge eating question because of survey procedures for schools selected for both the national and site-specific YRBS samples. Students in these schools completed only one questionnaire; when the site-specific questionnaire was administered, it might not have included all questions on the national YRBS, including the binge eating question (*13*). Among the 7,209 students who were administered the binge eating question, 311 did not provide a valid response, resulting in 6,898 students with valid binge eating data. Four additional health behaviors or experiences were examined: 1) social media use, categorized as a three-level variable (hourly or more, several times per day, once per day or less); 2) poor mental health, defined as reporting that their mental health was not good most of the time or always in the past 30 days; 3) persistent feelings of sadness or hopelessness, defined as reporting feelings of sadness or hopelessness for ≥2 weeks in a row in the past 12 months; and 4) seriously considered attempting suicide in the past 12 months. Distributions of response options for social media use, mental health, and suicide risk measures among students with valid binge eating data are provided (Supplementary Table 1).

**TABLE 1:** Questions, response options, and analytic coding for measures of binge eating, social media use, mental health, and suicide risk among high school students — Youth Risk Behavior Survey, United States, 2025

Variable	Question	Response option	Analytic coding
Binge eating in the past 30 days	During the past 30 days, on how many days did you eat an unusually large amount of food in a short period of time and experience a loss of control over how much you were eating or a feeling that you could not stop eating even when full?	0 days, 1 or 2 days, 3–5 days, 6–9 days, 10–19 days, 20–29 days, or all 30 days	≥3 days versus <3 days
Frequency of social media use	How often do you use social media?*	I do not use social media, a few times a month, about once a week, a few times a week, about once per day, several times per day, about once an hour, or more than once an hour	Hourly or more (about once an hour or more than once an hour) versus several times per day versus once per day or less (I do not use social media, a few times a month, about once a week, a few times a week, or about once per day)
Poor mental health	During the past 30 days, how often was your mental health not good? (Poor mental health includes stress, anxiety, and depression.)	Never, rarely, sometimes, most of the time, or always	Yes (most of the time or always) versus no (never, rarely, or sometimes)
Persistent feelings of sadness or hopelessness	During the past 12 months, did you ever feel so sad or hopeless almost every day for two weeks or more in a row that you stopped doing some usual activities?	Yes or no	Yes versus no
Seriously considered attempting suicide	During the past 12 months, did you ever seriously consider attempting suicide?	Yes or no	Yes versus no

* The 2025 National Youth Risk Behavior Survey questionnaire describes social media, “such as Instagram, TikTok, Snapchat, and X (formerly known as Twitter).”

Demographic variables included sex (female or male), grade (9, 10, 11, or 12), and race and ethnicity (American Indian or Alaska Native [AI/AN], Asian, Black or African American [Black], Hispanic or Latino [Hispanic], Middle Eastern or North African [ME/NA], Native Hawaiian or Pacific Islander, White, or multiracial [selected more than one racial category]). Persons of Hispanic origin might be of any race but are categorized as Hispanic; all racial groups are non-Hispanic.

### Analysis

Weighted prevalence estimates and corresponding 95% CIs were calculated for each response option for binge eating in the past 30 days. In addition, the weighted prevalence of binge eating on ≥3 days, with corresponding 95% CIs, was estimated overall and by demographic characteristics and the other health behaviors and experiences. Differences in the weighted prevalence of binge eating on ≥3 days by demographic characteristics were assessed using pairwise design-adjusted *t* tests. Logistic regression models with predicted marginals were used to estimate unadjusted and adjusted prevalence ratios (aPRs) and adjusted prevalence differences (aPDs) comparing the prevalence of binge eating on ≥3 days by social media use, mental health, and suicide risk measures. Separate models were fit for each social media use, poor mental health, persistent feelings of sadness or hopelessness, and suicide risk measure, controlling for sex, grade, and race and ethnicity. Sex-specific aPRs and aPDs also were estimated using models adjusted for grade and race and ethnicity. Unadjusted prevalence ratios and prevalence differences are provided (Supplementary Table 2). The prevalence of binge eating was compared across social media use categories, with once per day or less as the referent group. A secondary comparison examined students reporting social media use hourly or more compared with several times per day as the referent group. All prevalence estimates and measures of association used Taylor series linearization. Pairwise *t* tests and prevalence differences were considered statistically significant at p<0.05, and prevalence ratios were considered statistically significant when the 95% CI did not cross the null hypothesis value of 1.0. Corrections for multiple comparisons were not applied because the analyses were descriptive and hypothesis generating, and each statistical test addressed a distinct research question and was interpreted independently. Prevalence estimates with a denominator <30 were considered statistically unreliable and were suppressed; therefore, prevalence estimates for Native Hawaiian or Pacific Islander students were suppressed. All analyses were conducted with SAS-callable SUDAAN (version 11.0.4; RTI International) and accounted for the complex sampling design and weighting. Survey weights accounted for the complex sampling design and survey-level nonresponse but were not adjusted separately for item-specific missing data. In a sensitivity analysis to assess potential bias from missing binge eating data, distributions of sex, race and ethnicity, and grade were compared between students with and without available binge eating data; no statistically significant differences were observed.

## Results

In weighted analyses, 59.5% of students reported 0 days of binge eating, indicating that 40.5% of students reported binge eating on at least 1 day in the past 30 days (Table 2). In addition, 17.2% of students reported binge eating on ≥3 days (Table 3). Female students had a higher prevalence of reporting binge eating on ≥3 days compared with male students (18.6% versus 15.9%) (Table 3). No statistically significant differences were observed by grade level. Hispanic (20.5%) and Black (18.7%) students had a higher prevalence of binge eating on ≥3 days compared with White (14.6%) and ME/NA (9.9%) students. Multiracial (19.6%) students had a higher prevalence of binge eating on ≥3 days than ME/NA students.

**TABLE 2:** Prevalence of binge eating in the past 30 days among high school students, by number of days — Youth Risk Behavior Survey, United States, 2025*

Response option, no. of days	Unweighted no.	Weighted % (95% CI)
0 days	3,911	59.5 (57.5–61.5)
1 or 2 days	1,699	23.3 (21.9–24.7)
3–5 days	632	8.3 (7.4–9.3)
6–9 days	277	3.7 (3.1–4.4)
10–19 days	183	2.5 (2.1–3.1)
20–29 days	49	0.6 (0.4–0.9)
All 30 days	147	2.0 (1.6–2.5)
Missing	3,812	—

* N = 10,710 respondents. The total number (N) of students answering each question varied. Data might be missing because 1) students in schools selected for both national and site-specific YRBS samples completed only one questionnaire; therefore, when the site-specific questionnaire was administered, it might not have included all questions on the national YRBS; 2) the student did not answer the question; or 3) the response was set to missing because of an out-of-range response or logical inconsistency. Prevalence estimates in each category are calculated based on nonmissing data.

**TABLE 3:** Prevalence of binge eating on ≥3 days in the past 30 days among high school students, by demographic characteristics — Youth Risk Behavior Survey, United States, 2025*

Characteristic	% (95% CI)
**Overall**	**17.2 (15.8–18.7)**
**Sex** ^†^	
Female	18.6 (16.7–20.6)
Male	15.9 (14.4–17.6)
**Grade**	
9	16.7 (14.6–19.2)
10	17.3 (14.8–20.2)
11	16.8 (14.5–19.5)
12	17.6 (14.9–20.7)
**Race and ethnicity** ^§,¶^	
AI/AN	25.6 (11.5–47.5)
Asian	17.7 (13.8–22.3)
Black or African American**	18.7 (16.9–20.8)
Hispanic or Latino††	20.5 (18.2–23.1)
ME/NA^§§^	9.9 (4.7–19.7)
White	14.6 (12.9–16.6)
Multiracial	19.6 (14.9–25.4)

**Abbreviations:** AI/AN = American Indian or Alaska Native; ME/NA = Middle Eastern or North African.

* N = 6,898 respondents with valid responses for the binge eating item (of 10,710 participants in the 2025 national YRBS). The total number (N) of students answering each question varied. Data might be missing because 1) students in schools selected for both national and site-specific YRBS samples completed only one questionnaire; therefore, when the site-specific questionnaire was administered, it might not have included all questions on the national YRBS; 2) the student did not answer the question; or 3) the response was set to missing because of an out-of-range response or logical inconsistency. Prevalence estimates in each category are calculated based on nonmissing data.

^†^ Female students significantly differed from male students as determined by *t* test with Taylor series linearization (p<0.05).

^§^ Persons of Hispanic or Latino (Hispanic) origin might be of any race but are categorized as Hispanic; all racial groups are non-Hispanic.

^¶^ Prevalence estimates with a denominator <30 were considered statistically unreliable and were suppressed; therefore, estimates for Native Hawaiian or Pacific Islander students are not presented.

** Black or African American students significantly differed from ME/NA and White students as determined by *t* test with Taylor series linearization (p<0.05).

^††^ Hispanic students significantly differed from ME/NA and White students as determined by *t* test with Taylor series linearization (p<0.05).

^§§^ ME/NA students significantly differed from multiracial students as determined by *t* test with Taylor series linearization (p<0.05).

Among all students, those using social media hourly or more had a higher prevalence of binge eating on ≥3 days than did those using social media once per day or less (22.8% versus 12.9%; aPR = 1.70; aPD = 9.2) (Table 4). Students using social media hourly or more also had a higher prevalence of binge eating than did those using social media several times per day (22.8% versus 14.4%; aPR = 1.56; aPD = 8.1). The prevalence of binge eating on ≥3 days was higher among students who reported poor mental health than among those who did not (27.2% versus 14.0%; aPR = 1.95; aPD = 13.2) and higher among students who reported persistent feelings of sadness or hopelessness than among those who did not (25.4% versus 12.8%; aPR = 1.91; aPD = 11.8). Students who had seriously considered attempting suicide had a higher prevalence of binge eating on ≥3 days than those who had not (27.1% versus 15.3%; aPR = 1.72; aPD = 11.1). These associations between binge eating and social media use, mental health, and suicide risk were also observed among both female and male students (Table 4).

**TABLE 4:** Prevalence of binge eating on ≥3 days in the past 30 days among high school students, by measures of social media use, mental health, and suicide risk — Youth Risk Behavior Survey, United States, 2025*

Social media use, mental health, and suicide risk	Overall			Female			Male		
	% (95% CI)	aPR (95% CI)^†^	aPD^†^	% (95% CI)	aPR (95% CI)^§^	aPD^§^	% (95% CI)	aPR (95% CI)^§^	aPD^§^
Social media use^¶^									
Hourly or more	**22.8 (20.1–25.7)**	**1.70 (1.34–2.16)**	9.2**	24.9 (21.6–28.5)	1.67 (1.20–2.34)	9.9**	20.8 (17.4–24.7)	1.73 (1.31–2.27)	8.6**
Several times per day	**14.4 (12.9–16.0)**	**1.09 (0.87–1.36)**	**1.2**	15.0 (12.8–17.5)	1.02 (0.70–1.49)	0.3	13.6 (11.3–16.4)	1.16 (0.83–1.63)	1.9
Once per day or less	**12.9 (10.6–15.6)**	**Ref**	**Ref**	15.1 (11.1–20.3)	Ref	Ref	11.6 (9.1–14.7)	Ref	Ref
**Poor mental health**									
Yes (most of the time or always)	**27.2 (23.8–30.9)**	**1.95 (1.68–2.27)**	13.2**	26.8 (23.1–30.9)	1.87 (1.57–2.21)	12.6**	28.2 (23.3–33.6)	2.06 (1.66–2.56)	14.2**
No (never, rarely, or sometimes)	**14.0 (12.7–15.3)**	**Ref**	**Ref**	14.7 (12.9–16.7)	Ref	Ref	13.4 (11.8–15.1)	Ref	Ref
**Persistent feelings of sadness or hopelessness**									
Yes	**25.4 (23.1–27.8)**	**1.91 (1.66–2.18)**	11.8**	26.4 (24.0–29.0)	2.05 (1.75–2.39)	13.3**	23.6 (20.0–27.5)	1.75 (1.42–2.17)	9.9**
No	**12.8 (11.4–14.4)**	**Ref**	**Ref**	12.5 (10.7–14.7)	Ref	Ref	13.0 (11.3–15.0)	Ref	Ref
**Seriously considered attempting suicide**									
Yes	**27.1 (23.0–31.6)**	**1.72 (1.45–2.05)**	11.1**	27.5 (22.5–33.2)	1.64 (1.32–2.03)	10.6**	26.4 (21.0–32.6)	1.82 (1.43–2.32)	11.7**
No	**15.3 (13.9–16.8)**	**Ref**	**Ref**	16.6 (14.7–18.7)	Ref	Ref	14.2 (12.7–15.9)	Ref	Ref

**Abbreviations:** aPD = adjusted prevalence difference; aPR = adjusted prevalence ratio; Ref = referent.

* N = 6,898 respondents with valid responses for the binge eating item (of 10,710 participants in the 2025 national YRBS). The total number (N) of students answering each question varied. Data might be missing because 1) students in schools selected for both national and site-specific YRBS samples completed only one questionnaire; therefore, when the site-specific questionnaire was administered, it might not have included all questions on the national YRBS; 2) the student did not answer the question; or 3) the response was set to missing because of an out-of-range response or logical inconsistency. Prevalence estimates and analyses in each category are calculated based on nonmissing data.

**^†^** Adjusted for sex, grade, and race and ethnicity.

^§^ Adjusted for grade and race and ethnicity.

^¶^ Once per day or less was the referent group for the primary comparisons shown. A secondary comparison (not shown) examined students reporting social media use hourly or more compared with several times per day, with several times per day as the referent group: overall aPR = 1.56 (95% CI = 1.33–1.84); overall aPD = 8.1 (p<0.001); female students aPR = 1.64 (95% CI = 1.35–1.99); female students aPD = 9.6 (p<0.001); male students aPR = 1.48 (95% CI = 1.11–1.99); male students aPD = 6.6 (p = 0.01).

** Statistically significant as determined by a pairwise difference from the adjusted logistic regression model with predicted marginal proportions (p<0.05).

## Discussion

This report provides the first national prevalence estimate of binge eating behavior from a representative sample of U.S. high school students. In 2025, 40.5% of students reported binge eating on at least 1 day, and 17.2% reported binge eating on ≥3 days in the past 30 days. The prevalence of binge eating on ≥3 days was higher among female students (18.6%) than among male students (15.9%). Racial and ethnic differences were also observed for binge eating on ≥3 days, with Black and Hispanic students reporting higher prevalences than White and ME/NA students and multiracial students reporting a higher prevalence than ME/NA students. However, comparisons involving ME/NA students should be interpreted with caution because the ME/NA estimate was imprecise due to the small sample size. Furthermore, using social media hourly or more, having poor mental health, having persistent sadness or hopelessness, and having seriously considered attempting suicide were associated with a higher prevalence of binge eating on ≥3 days, and these associations were observed among both female and male students.

The proportion of students reporting binge eating on ≥3 days might be concerning, given reported associations of binge eating with increased risk for eating disorders and negative health outcomes (*5*,*7*,*8*). Although binge eating prevalence was lower among male than female students, prevalence among male students was still notable, and associations with social media use, mental health, and suicide risk were observed among both female and male students. These findings suggest that male students might be an important population to consider in prevention and early identification efforts, along with female students, particularly given previous studies indicating that disordered eating among male youths could be underrecognized (*14*). In addition, the prevalence of binge eating on ≥3 days was 18.7% among Black students, 20.5% among Hispanic students, and 25.6% among AI/AN students; however, the estimate for AI/AN students was imprecise, and larger sample sizes are needed. These findings could be considered in the context of concerns that eating disorders might be underrecognized in some racial and ethnic groups and suggest the importance of greater attention to binge eating and disordered eating risk across different subpopulations (*15*).

The findings on binge eating and social media use are consistent with previous research suggesting that frequent social media use is associated with binge eating, although studies that have focused on school-aged populations remain limited (*10*). In this report, the prevalence of binge eating on ≥3 days was higher among students who reported social media use hourly or more than among those who reported use several times per day or once per day or less, whereas the prevalence did not differ significantly between the two lower-use groups. Although the underlying mechanisms of this pattern remain unclear, research suggests that exposure to appearance- and diet-related content, comparisons with others’ appearances, and internalization of idealized body standards might contribute to body dissatisfaction and subsequent binge eating risk (*10*,*16*). These dynamics might be of particular concern during adolescence, given their substantial exposure to social media during a period of physical and social development (*10*,*16*).

Findings regarding the association between binge eating, mental health, and suicide risk are consistent with previous research. For example, a study that analyzed 2023 Alaska YRBS data demonstrated statistically significant associations between binge eating and both depressive symptoms and suicidal ideation among high school students (*8*). These findings also align with other research reporting associations between youth binge eating and co-occurring mental health conditions, including depression, anxiety, and suicide risk (*7*). One proposed hypothesis is that some youths might use binge eating to regulate their emotions and then experience shame or guilt afterward; however, the direction and timing of the association between binge eating and mental health remain uncertain (*7*,*17*).

The findings in this report highlight the importance of continued emphasis on disordered eating prevention and intervention among youths by national, state, and local public health and education professionals. Efforts to address disordered eating among youths could benefit from coordination with families, health care providers, and mental health clinicians to support prevention, early identification, and treatment (*18*). Resources from the Substance Abuse and Mental Health Services Administration’s National Center of Excellence for Eating Disorders (https:/nceedus.org), including eating disorder training and toolkits for health care providers and resources for families, could support these efforts.

Schools might be well-positioned to complement these efforts through prevention and early identification, particularly when staff members are trained and receive support through school resources and planning (*18*). Such efforts could be embedded within school health offices, health education initiatives, and broader school environments and policies. Existing frameworks for improving health education and school environments could help guide implementation. For example, the Whole School, Whole Community, Whole Child framework (https://www.cdc.gov/whole-school-community-child/about/index.html) could be used to address a wide range of health issues through schools. In addition, CDC has two school health programs, What Works in Schools (https://www.cdc.gov/healthy-youth/what-works-in-schools/index.html) and the Healthy Schools Program (https://www.cdc.gov/healthy-schools/about/index.html), that address health and physical education, connection to health services and supporting students with health conditions, healthy school environments, and family and community engagement. Programs and models such as these provide frameworks that could be adapted to incorporate prevention and recognition of disordered eating. Specifically, school staff members, including nurses, counselors, psychologists, teachers, and coaches, could consider using nonstigmatizing language in nutrition education, discussing media literacy and the use of enhanced images, fostering an environment inclusive of all body sizes, and supporting early identification and referral (*19*). These approaches were not evaluated in this study.

Given the associations between binge eating, poor mental health, and suicide risk described in this report, strategies to reduce mental distress and suicide risk, such as those described in the National Strategy for Suicide Prevention (https://www.hhs.gov/programs/prevention-and-wellness/mental-health-substance-use-disorder/national-strategy-suicide-prevention/index.html) and CDC’s Suicide Prevention Resource for Action (https://www.cdc.gov/suicide/php/public-health-strategy/index.html), could also be integrated into school and community prevention efforts.

The inclusion of the binge eating question on the 2025 national YRBS demonstrates how collaboration between CDC and partners in adolescent health and eating disorder prevention can guide surveillance priorities. A 2024 report from the Eating Disorder Public Health Surveillance Working Group recommended assessment of disordered eating in YRBS (*20*); external collaborators on this report participated in this working group. This partner input helped guide inclusion of binge eating as a surveillance priority and the collection of national data on disordered eating behaviors among high school students. CDC supports nongovernmental organizations that in turn support state education and health agencies in improving the health and well-being of students and staff through professional development and technical assistance, dissemination, and partnerships focused on disordered eating. These activities highlight the potential importance of partnerships among researchers, public health agencies, school districts, and organizations focused on eating disorder prevention in strengthening surveillance and supporting school-based prevention and early identification efforts across U.S. schools.

## Limitations

General limitations for YRBS are available in the overview report of this supplement (*13*). The findings in this report are subject to at least five additional limitations. First, the majority of missing binge eating data were from students who were not administered the question, which might limit generalizability. However, students with and without available binge eating data did not differ significantly by sex, race and ethnicity, or grade. Second, the cross-sectional design and differing recall periods across survey questions (e.g., 30 days for binge eating and poor mental health versus 12 months for persistent sadness or hopelessness and considering suicide) limit causal interpretation, including the direction and timing of the associations among these measures. Third, potential confounders not captured in YRBS, such as socioeconomic status, could independently influence social media use, mental health outcomes and suicide risk, and binge eating. Fourth, the social media measure captured frequency of use but did not assess duration, exposure to specific content, platform, or type of engagement, which might be more directly related to binge eating. Finally, measuring the complex psychological and behavioral aspects of binge eating with a single survey question is challenging. Some students might have confused occasional overeating with binge eating, which could have resulted in overestimates of prevalence. However, cognitive testing of the item suggested that most students understood the question and considered the loss-of-control component when responding.

## Future Directions

The prevalence of binge eating, observed differences across demographic groups, and associations with social media use, mental health, and suicide risk highlight the potential importance of continued surveillance to monitor trends, identify disproportionately affected groups, and guide prevention efforts. Future research could examine the underlying mechanisms linking binge eating and social media use, mental health, and suicide risk by sex, race and ethnicity, and other demographic characteristics and assess whether existing evidence-based interventions require adaptation for specific populations.

These new representative data indicate that binge eating might be common among adolescents; however, translating these data into action might require addressing existing implementation gaps. Despite increasing awareness of disordered eating among youths, previous studies have noted limited public health engagement in eating disorder prevention and a potential need to broaden prevention approaches beyond individually focused interventions (*21*). Implementation of school-based interventions for eating disorder prevention and early identification might be supported by staff training, resource development, and planning (*18*). In addition, introducing weight-inclusive approaches within school environments where weight-normative programs and practices remain common might create uncertainty for school staff members about how to apply weight-inclusive guidance, particularly when training and implementation resources are limited (*22*). Without such supports, school staff members might face barriers to addressing disordered eating among students. For example, a survey of school psychologists practicing in K–12 public schools across the United States found that respondents reported limited preparation and knowledge regarding supports for students with disordered eating, and many had sought additional training; however, these findings might not generalize to other school staff members or U.S. schools overall (*23*).

## Conclusion

Binge eating was reported by a notable proportion of U.S. high school students, with higher prevalence among female than male students, Black and Hispanic students than White and ME/NA students, and multiracial students than ME/NA students. In addition, students who reported using social media hourly or more, poor mental health, persistent sadness or hopelessness, or having seriously considered attempting suicide had a higher prevalence of reporting binge eating behavior on ≥3 days. The findings suggest the importance of continued data collection and more comprehensive surveillance of disordered eating. Such efforts could include assessing behaviors beyond binge eating and monitoring associated factors and trends over time. Findings also might support continued attention to disordered eating prevention and intervention, including comprehensive approaches that address factors associated with disordered eating.

## Footnotes

* 45 C.F.R. part 46.114; 21 C.F.R. part 56.114.
